# The secretome atlas of two mouse models of progeria

**DOI:** 10.1111/acel.13952

**Published:** 2023-08-10

**Authors:** Diego Quintana‐Torres, Alejandra Valle‐Cao, Pablo Bousquets‐Muñoz, Sandra Freitas‐Rodríguez, Francisco Rodríguez, Alejandro Lucia, Carlos López‐Otín, Alejandro López‐Soto, Alicia R. Folgueras

**Affiliations:** ^1^ Departamento de Bioquímica y Biología Molecular, Facultad de Medicina Instituto Universitario de Oncología del Principado de Asturias (IUOPA), Universidad de Oviedo Oviedo Spain; ^2^ Instituto de Investigación Sanitaria del Principado de Asturias (ISPA) Oviedo Spain; ^3^ CIBER of Frailty and Healthy Aging (CIBERFES) and Instituto de Investigación 12 de Octubre (i+12) Madrid Spain; ^4^ Faculty of Sport Sciences Universidad Europea Madrid Spain

**Keywords:** aging, aging clock, HGPS, progeria, proteomics, secretome

## Abstract

Hutchinson‐Gilford progeria syndrome (HGPS) is a rare genetic disease caused by nuclear envelope alterations that lead to accelerated aging and premature death. Several studies have linked health and longevity to cell‐extrinsic mechanisms, highlighting the relevance of circulating factors in the aging process as well as in age‐related diseases. We performed a global plasma proteomic analysis in two preclinical progeroid models (*Lmna* ^*G609G/G609G*^ and *Zmpste24* ^*−/−*^ mice) using aptamer‐based proteomic technology. Pathways related to the extracellular matrix, growth factor response and calcium ion binding were among the most enriched in the proteomic signature of progeroid samples compared to controls. Despite the global downregulation trend found in the plasma proteome of progeroid mice, several proteins associated with cardiovascular disease, the main cause of death in HGPS, were upregulated. We also developed a chronological age predictor using plasma proteome data from a cohort of healthy mice (aged 1–30 months), that reported an age acceleration when applied to progeroid mice, indicating that these mice exhibit an “old” plasma proteomic signature. Furthermore, when compared to naturally‐aged mice, a great proportion of differentially expressed circulating proteins in progeroid mice were specific to premature aging, highlighting secretome‐associated differences between physiological and accelerated aging. This is the first large‐scale profiling of the plasma proteome in progeroid mice, which provides an extensive list of candidate circulating plasma proteins as potential biomarkers and/or therapeutic targets for further exploration and hypothesis generation in the context of both physiological and premature aging.

## Graphical abstract

Large‐scale profiling of the plasma proteome of two mouse models of Hutchinson‐Gilford Progeria Syndrome was conducted by means of an aptamer‐based proteomic technology (SomaScan Assay). This assay revealed significant alterations in proteins related to the extracellular matrix, growth factor response and calcium ion binding, as well as an upregulation in certain proteins associated with cardiovascular disease, among others. A “proteomic clock” was developed using plasma proteome data from healthy mice during aging, revealing an age acceleration when applied to progeroid mice.

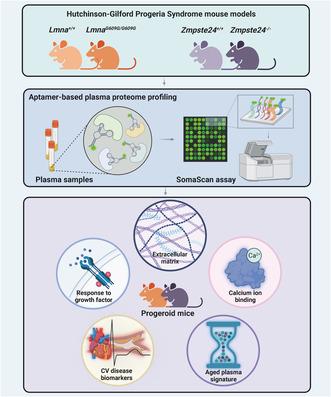

**Abbreviations**
CVD: cardiovascular diseases
DE: differentially expressed
ECM: extracellular matrix
EDTA: ethylenediaminetetraacetic acid
ELISA: enzyme‐linked immunosorbent assay
FC: fold change
FDR: false discovery rate
FTI: farnesyl‐transferase inhibitor
GH: growth hormone
GO: Gene Ontology
HGPS: Hutchinson‐Gilford progeria syndrome
LASSO: least absolute shrinkage and selection operator
PCA: principal component analysis
RFU: relative fluorescence units

## 1 INTRODUCTION

Aging is a multifactorial process that leads to a progressive decay in biological function owing to the accumulation of cellular and macromolecular damage. This organismal dysfunction is in turn a main risk factor for several noncommunicable conditions, including cardiovascular diseases (CVD), neurodegenerative disorders and various types of cancer (Campisi et al., 2019; Franceschi et al., 2018). Thus, the new worldwide demographic scenario, characterized by an aging population that is more prone to suffering from chronic diseases, has become a major health and social challenge. Consequently, a great effort is being made to identify the molecular determinants that drive the aging process. This will allow to define potential therapeutic interventions to reduce the susceptibility to disease of older people and thereby to improve not only their lifespan, but also their healthspan.

Premature aging disorders, also known as progeroid syndromes, are a group of rare diseases characterized by the onset from early childhood of clinical features that resemble physiological aging, thereby making them an interesting model for studying the biology underlying such a complex process (Carrero et al., 2016). Hutchinson‐Gilford progeria syndrome (HGPS) is one of the most widely studied premature aging disorders. Patients with HGPS suffer from growth impairment, lipodystrophy, bone defects, muscle wasting and CVD, among others, which ultimately shorten their life expectancy to an average of 14.6 years (Gordon, Shappell, et al., 2018; Hennekam, 2006). Most patients with HGPS carry a dominant synonymous mutation (c.1824C>T, p.G608G) in exon 11 of the *LMNA* gene, which encodes both lamin A and lamin C nuclear envelope proteins. As a consequence of this point mutation, a cryptic splice donor site is activated in the *LMNA* transcript, leading to the accumulation of an aberrant, unprocessed lamin A isoform called “progerin”. This mutant protein lacks 50 amino acids, including a cleavage site for the zinc metallopeptidase STE24 (ZMPSTE24), which removes the farnesylated C‐terminal end of prelamin A during its maturation process. Thus, progerin is a permanently farnesylated protein that perturbs the nuclear architecture and functionality, ultimately triggering the molecular and cellular alterations which result in the progeroid phenotype (Gordon et al., 2014; Shin & Worman, 2022).

The generation of mouse models that recapitulate many of the clinical manifestations of HGPS, by mimicking the point mutation found in most affected patients (*Lmna* ^*G609G/G609G*^ mice) (Osorio et al., 2011) or by knocking‐out *Zmpste24* gene (*Zmpste24* ^*−/−*^ mice) (Bergo et al., 2002; Pendás et al., 2002), has become an invaluable tool to understand the mechanisms that drive the aging process and to test potential therapeutic interventions aimed at improving progeroid features and age‐related diseases. To date, several treatment strategies targeting different aspects of the disease, such as the generation of progerin (Koblan et al., 2021; Osorio et al., 2011; Puttaraju et al., 2021; Santiago‐Fernández et al., 2019) or its nuclear accumulation and downstream deleterious effects (Harhouri et al., 2018; Lai & Wong, 2020; Macicior et al., 2021), have shown an amelioration of the progeroid phenotype and an increased lifespan in preclinical mouse models. As a result of this scientific effort, the first drug approved for the treatment of progeria, lonafarnib, a farnesyl‐transferase inhibitor (FTI), has increased the survival of patients by almost 5 years (Gordon et al., 2023). Despite this promising achievement, further work is needed to accomplish a permanent cure for this devastating disease.

A hallmark of both pathological and physiological aging is the alteration of intercellular communication, a progressive phenomenon which ultimately perturbs body homeostasis (López‐Otín et al., 2023). Indeed, the accumulation of molecular and cellular damage inherent in the aging process is reflected in the secretome released, which exerts both local and systemic key biological functions. Hence, over the last years, a number of interventions aimed to manipulating circulating factors have been explored in order to restrain age‐dependent organismal deterioration. Of note, the administration of both “old” and “young” plasma has been demonstrated to induce or ameliorate diverse aging phenotypes in mice, highlighting the relevance of certain systemic factors as powerful pro‐ or anti‐aging effectors (Ma et al., 2022; Rebo et al., 2016; Villeda et al., 2014). Accordingly, these findings have prompted the search for circulating proteins that may become potential therapeutic targets and/or biomarkers that contribute to intervene the aging process and age‐related diseases (Lehallier et al., 2019; Sathyan et al., 2020; Tanaka et al., 2018; Williams et al., 2019). Given the inherent organismal deterioration associated with premature aging syndromes, we hypothesized that this molecular and cellular damage may be, at least, partially recapitulated at the secretome level, where certain blood factors might also play a role in the development, maintenance, and progress of the progeroid phenotype. Hopefully, this scenario would allow the identification of potential HGPS biomarkers or therapeutic targets. Therefore, in this study, we conducted, to the best of our knowledge, the first global profiling of the plasma proteome of two progeroid mouse models (*Lmna* ^*G609G/G609G*^ and *Zmpste24* ^*−/−*^) to identify systemic factors altered in premature aging syndromes. To this end, we used the SomaScan Assay (SomaLogic Inc.), an aptamer‐based proteomic technology capable of recognizing and, simultaneously, measuring the levels of thousands of proteins with high specificity. At the same time, we sought to identify altered proteins in common with physiological aging and develop a “proteomic clock” from public data to predict the biological age of these models.

## 2 RESULTS

### 2.1 Plasma proteome profiling of progeroid mice

It has been demonstrated that the composition of the plasma proteome changes throughout life in both humans and mice (Lehallier et al., 2019). To gain insight into this phenomenon, we analyzed the plasma proteome of two progeroid mouse models by an aptamer‐based global approach. For this purpose, we performed a SomaScan Assay to analyze the protein composition of plasma samples from 4.5‐ to 5‐month‐old *Lmna* ^*+/+*^ and *Lmna* ^*G609G/G609G*^ mice, and ~6.5‐month‐old *Zmpste24* ^*+/+*^ and *Zmpste24* ^*−/−*^ mice (*n* = 10 mice per group). For each group, 5 male and 5 female mice were included. Of note, *Zmpste24* ^*−/−*^ mice live longer than *Lmna* ^*G609G/G609G*^ mice (Bárcena et al., 2018).

**FIGURE 1:**
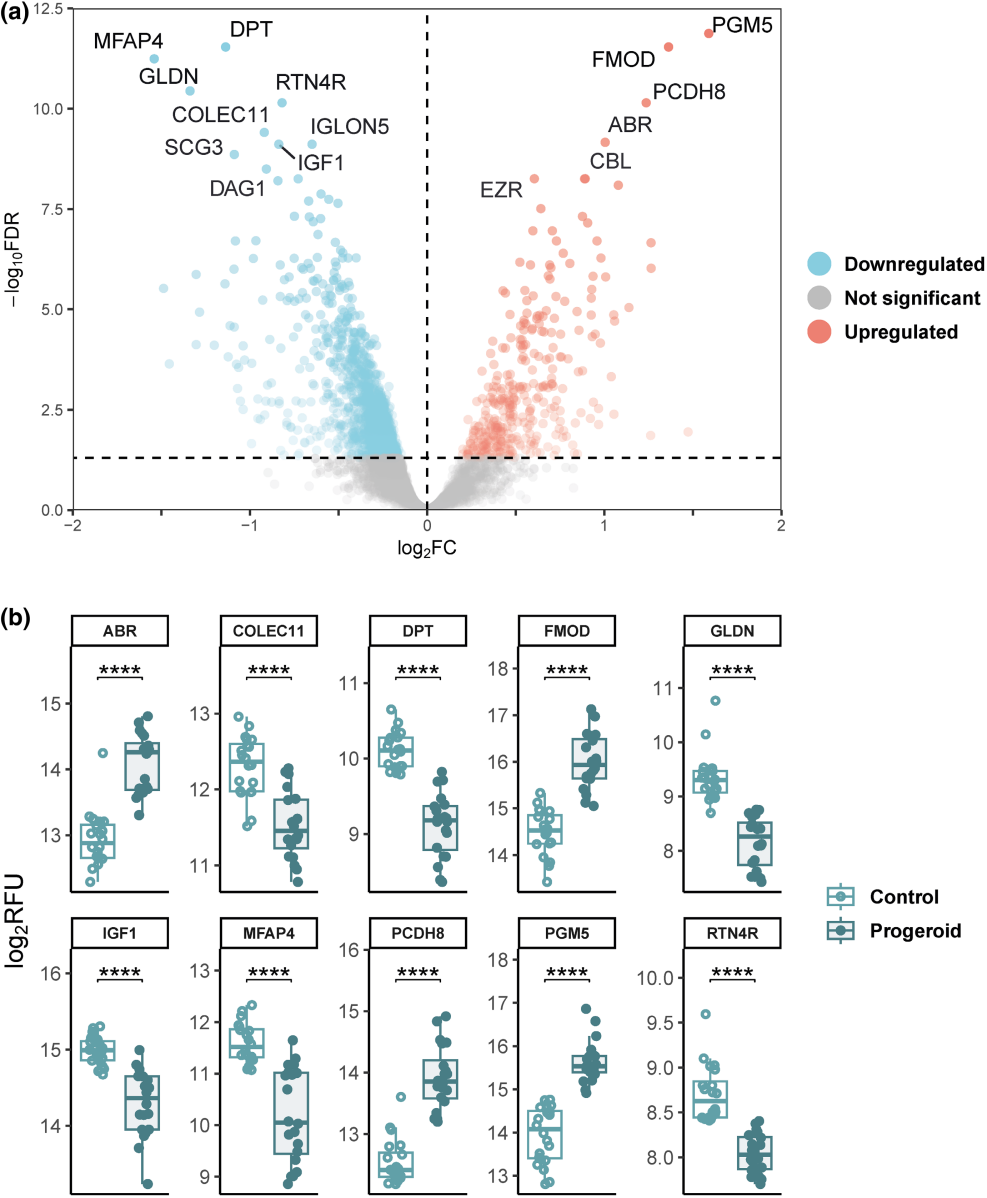
Progeroid plasma proteome characterization. (a) Volcano plot representing the plasma protein differences (*n* = 7291 aptamers) in progeroid mice versus controls. Down‐ (FDR <0.05, log_2_(fold change) <0) and upregulated proteins (FDR <0.05, log_2_FC >0) are shown as blue and red points, respectively. The *x*‐axis represents the magnitude of the difference expressed as log_2_FC whereas the *y*‐axis represents significance level expressed as −log_10_FDR. (b) Boxplots of protein levels expressed as log_2_RFU for each biological replicate for the top 10 most significant differentially expressed proteins between control and progeroid mice. ABR, active breakpoint cluster region‐related protein; CBL, Cbl proto‐oncogene; COLEC11, collectin subfamily member 11; DAG1, dystroglycan 1; DPT, dermatopontin; EZR, ezrin; FMOD, fibromodulin; GLDN, gliomedin; IGF1, insulin‐like growth 1; IGLON5, IgLON family member 5, Ig‐like domain‐containing protein; MFAP4, microfibril associated protein 4; PCDH8, protocadherin 8; PGM5, phosphoglucomutase 5; RTN4R, reticulon 4 receptor; SCG3, secretogranin III.

Using version 4.1 of the SomaScan Assay, we successfully measured the levels of 7291 aptamers targeting 6388 unique proteins of interest. An initial exploratory data analysis showed an unexpected significant effect for the cohort (Figure S1). After adjusting for the different covariates (mouse model, sex, and cohort) and fitting a linear model for each aptamer, we identified 2475 proteins that were differentially expressed (DE) in control (*n* = 20) and progeroid mice (*n* = 20) (False Discovery Rate [FDR] <0.05). Among these, 2119 were downregulated and 356 were upregulated (Figure 1a). Remarkably, most of the proteins whose plasma concentration differed between control and progeroid mice were downregulated (85.6%), with only a small fraction (14.4%) upregulated.

The top 10 most significant proteins DE in progeroid versus wild‐type mice included proteins involved in cytoskeleton organization (active breakpoint cluster region‐related protein [ABR] and reticulon 4 receptor [RTN4R]) and regulators of the extracellular matrix structure (phosphoglucomutase 5 [PGM5], fibromodulin [FMOD], dermatopontin [DPT], and microfibril associated protein 4 [MFAP4]). Proteins related to cell adhesion (protocadherin 8 [PCDH8]), apoptosis and innate immunity (collectin subfamily member 11 [COLEC11]), and Ranvier node formation in the nervous system (gliomedin [GLDN]) were also found in this group (Figure 1b and Table 1). Likewise, insulin‐like growth factor 1 (IGF‐1), the primary regulator of somatic growth, was one of the most downregulated proteins in progeroid mice (Figure 1b). Additional tests regarding sex‐specific effects in progeroid mice (sex:genotype interaction term in the linear model) were carried out, but significance (FDR <0.05) was only met for two proteins (Figure S2a and Table 2).

**TABLE 1:** Top 10 most significant differentially expressed proteins in progeroid mice.

Gene symbol	UniProt ID	Aptamer	log_2_FC	FDR
*PGM5*	Q15124	seq.25117.17	1.59	1.33E‐12
*DPT*	Q07507	seq.4979.34	−1.14	2.88E‐12
*FMOD*	Q06828	seq.6367.66	1.36	2.88E‐12
*MFAP4*	P55083	seq.5636.10	−1.54	5.69E‐12
*GLDN*	Q6ZMI3	seq.20591.48	−1.34	3.60E‐11
*RTN4R*	Q9BZR6	seq.5105.2	−0.82	7.10E‐11
*PCDH8*	O95206	seq.6280.11	1.24	7.10E‐11
*COLEC11*	Q9BWP8	seq.4430.44	−0.92	3.90E‐10
*ABR*	Q12979	seq.21958.4	1.00	6.87E‐10
*IGF1*	P05019	seq.2952.75	−0.84	7.66E‐10

**TABLE 2:** Proteins with a significant sex:genotype interaction in progeroid mice.

Comparison	Gene symbol	UniProt ID	Aptamer	FDR
*Progeroid*	*SFRP4*	Q6FHJ7	seq.17447.52	8.54E‐03
*Progeroid*	*TMUB1*	Q9BVT8	seq.25255.4	9.20E‐03
*Lmna*	*TMUB1*	Q9BVT8	seq.25255.4	1.26E‐02
*Lmna*	*PLSCR3*	Q9NRY6	seq.11407.57	1.26E‐02
*Lmna*	*SFRP4*	Q6FHJ7	seq.17447.52	3.26E‐02
*Zmpste24*	*SUMF2*	Q8NBJ7	seq.6069.71	4.42E‐03
*Zmpste24*	*FURIN*	P09958	seq.6276.16	6.79E‐03
*Zmpste24*	*NAP1L1*	P55209	seq.13636.20	1.12E‐02
*Zmpste24*	*RABEP1*	Q15276	seq.24417.22	1.12E‐02
*Zmpste24*	*HSPA1A*	P0DMV8	seq.10721.76	1.12E‐02
*Zmpste24*	*GPCPD1*	Q9NPB8	seq.12786.61	1.64E‐02
*Zmpste24*	*DNASE1L2*	Q92874	seq.6324.11	1.64E‐02
*Zmpste24*	*HSPA1A*	P0DMV8	seq.6563.78	1.64E‐02
*Zmpste24*	*PIP4K2B*	P78356	seq.18232.42	2.37E‐02
*Zmpste24*	*UBE2N*|*UBE2V1*	P61088|Q13404	seq.21747.114	2.37E‐02
*Zmpste24*	*HSPA1A*	P0DMV8	seq.10803.22	3.04E‐02
*Zmpste24*	*PRDX3*	P30048	seq.8358.30	4.39E‐02
*Zmpste24*	*OSBPL1A*	Q9BXW6	seq.25266.14	4.39E‐02
*Zmpste24*	*NACA*	Q13765	seq.3854.24	4.39E‐02
*Zmpste24*	*NONO*	Q15233	seq.24910.18	4.39E‐02
*Zmpste24*	*PSPC1*	Q8WXF1	seq.24948.79	4.39E‐02

The high prevalence of downregulated proteins in the plasma of progeroid mice suggests that a broad range of cellular and molecular processes may be impacted by premature aging.

### 2.2 Commonalities and differences in the plasma proteome of two murine models of premature aging

Both *Lmna* ^*G609G/G609G*^ and *Zmpste24* ^*−/−*^ mice are highly valuable models for studying HGPS and share many phenotypic characteristics. Nevertheless, model differences may account for some distinct molecular signatures that would be reflected in their plasma proteome profile. To evaluate this hypothesis, we performed model‐specific analyses by comparing each progeroid model to its corresponding control.

**FIGURE 2:**
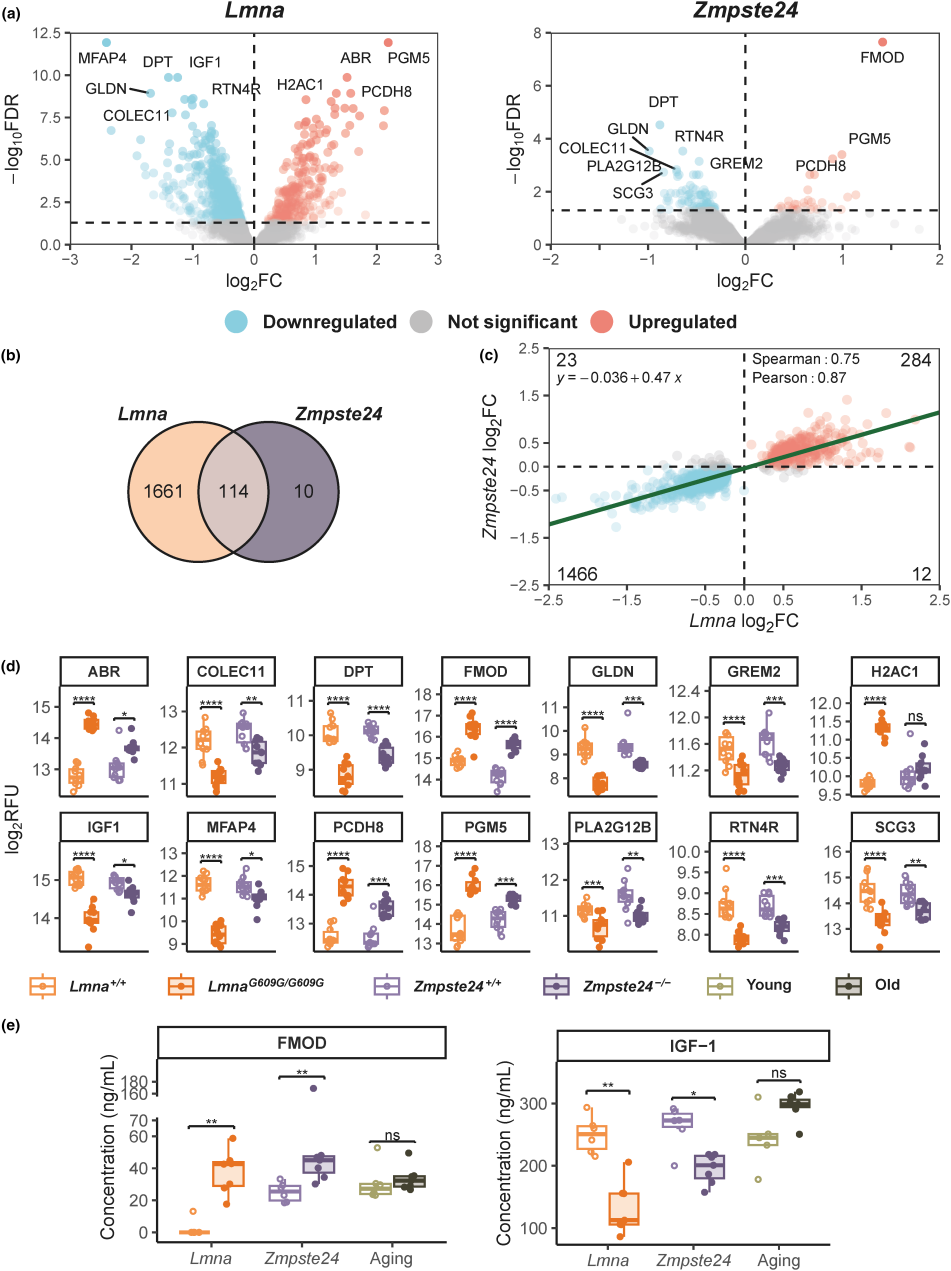
Plasma proteome profiles within *Lmna* ^*G609G/G609G*^ and *Zmpste24* ^*−/−*^ mouse models display a similar trend, with differences versus the corresponding controls further exacerbated in *Lmna* ^*G609G/G609G*^ mice. (a) Volcano plots representing the plasma protein differences (*n* = 7291 aptamers) in *Lmna* ^*G609G/G609G*^ (left) and *Zmpste24* ^*−/−*^ mice (right) compared to their wild‐type controls. Down‐ (FDR <0.05, log_2_FC <0) and upregulated proteins (FDR <0.05, log_2_FC >0) are shown as blue and red points, respectively. The *x*‐axis represents the magnitude of difference expressed as log_2_FC whereas the *y*‐axis represents significance level expressed as ‐log_10_FDR. (b) Venn diagram showing the overlap between differentially expressed aptamers in *Lmna* ^*G609G/G609G*^ and *Zmpste24* ^*−/−*^ mice compared to their wild‐type controls. (c) Scatter plot displaying the log_2_FC of *Lmna* ^*G609G/G609G*^ and *Zmpste24* ^*−/−*^ in each axis. The result of a linear regression model fitted to the data is shown as a green line. For this model, Spearman and Pearson correlation coefficients are provided. (d) Boxplots of protein levels expressed as log_2_RFU for each biological replicate for the top 10 most significant DE proteins within *Lmna* ^*G609G/G609G*^ and *Zmpste24* ^*−/−*^ mouse models compared to their respective wild‐type controls. A total of 14 unique proteins shared among the top 10 for both models are displayed. (e) Plasma protein concentrations of FMOD and IGF‐1, measured by ELISA, of *Lmna* ^*+/+*^ (*n* = 6), *Lmna* ^*G609G/G609G*^ (*n* = 7), *Zmpste24* ^*+/+*^ (*n* = 6), *Zmpste24* ^*−/−*^ (FMOD: *n* = 6, IGF‐1: *n* = 7), young (FMOD: *n* = 6, IGF‐1: *n* = 5) and old (*n* = 7) mice. ABR, active breakpoint cluster region‐related protein; COLEC11, collectin subfamily member 11; DPT, dermatopontin; FMOD, fibromodulin; GLDN, gliomedin; GREM2, gremlin 2, DAN family BMP antagonist; H2AC1, H2A clustered histone 1; IGF‐1, insulin‐like growth 1; MFAP4, microfibril associated protein 4; PCDH8, protocadherin 8; PGM5, phosphoglucomutase 5; PLA2G12B, phospholipase A2 group XIIB; RTN4R, reticulon 4 receptor; SCG3, secretogranin III.

The analysis conducted on *Lmna* ^*G609G/G609G*^ mice (*n* = 10) revealed that among the 7291 aptamers included in the analysis, 1775 were DE compared to their wild‐type controls (*n* = 10) (FDR <0.05) (Figure 2a, left and Table S1). Specifically, 295 aptamers showed higher plasma levels, while the remaining 1480 were downregulated (Figure 2a, left). The analysis by sex only yielded 3 proteins (phospholipid scramblase 3 [PLSCR3], secreted frizzled related protein 4 [SFRP4] and transmembrane and ubiquitin‐like domain‐containing 1 [TMUB1]) showing a significant interaction between sex and genotype (Figure S2b and Table 2). Interestingly, when analyzing the plasma proteome composition of *Zmpste24* ^*−/−*^ (*n* = 10) versus their controls (*Zmpste24* ^*+/+*^ [*n* = 10]) using the same criteria as for *Lmna* ^*G609G/G609G*^ mice, only 124 of the 7291 aptamers analyzed were identified as DE in *Zmpste24* ^*−/−*^ mice (Figure 2a, right and Table S2). Among these DE aptamers, 30 showed higher plasma levels, while 94 were downregulated (Figure 2a, right). The analysis of these data by sex revealed 14 unique proteins (16 aptamers) with a significant positive or negative synergy between genotype and sex (Figure S2c and Table 2).

Despite the relatively low number of DE aptamers found in *Zmpste24* ^*−/−*^ mice compared to *Lmna* ^*G609G/G609G*^ mice, further inspection of the model‐specific comparisons showed that more than 90% of the significant DE aptamers in *Zmpste24* ^*−/−*^ mice were shared with *Lmna* ^*G609G/G609G*^ mice (Figure 2b). These results are in agreement with the strong, shared effect on the plasma protein levels observed in our first global analysis when both models were analyzed together. On this basis, we wondered whether the private DE aptamers found in *Lmna* ^*G609G/G609G*^ mice were indeed specific to this mouse model or whether, by contrast, the phenotype was simply more exacerbated in *Lmna* ^*G609G/G609G*^ than in *Zmpste24* ^*−/−*^ mice. To address this question, we modeled by linear regression the fold‐change values within the statistically significant proteins in any of the model‐specific comparisons. Interestingly, we found a high correlation between *Lmna* ^*G609G/G609G*^ and *Zmpste24* ^*−/−*^ mice (Pearson: 0.87, Spearman: 0.75) and a regression coefficient of 0.47 (Figure 2c). Altogether, our results suggest that, although the plasma proteome might undergo similar molecular alterations in both mouse models of progeria, these changes are more exacerbated in *Lmna* ^*G609G/G609G*^ mice. As a representative example of this finding are the top 10 most significant DE aptamers in any of the model‐specific comparisons, which show, in general, greater differences in *Lmna* ^*G609G/G609G*^ than in *Zmpste24* ^*−/−*^ mice, compared to their relevant wild‐type controls (Figure 2d). We next assayed by enzyme‐linked immunosorbent assay (ELISA) the plasma levels of FMOD and IGF‐1 to validate the results obtained with SomaScan Assay. Also, plasma samples obtained from naturally‐aged mice were included in these experiments (Figure 2e). In accordance with the SomaScan results, FMOD levels were significantly upregulated in the plasma of both progeroid mouse models, with no marked changes observed in old versus young mice. Interestingly, an opposite behavior was observed between physiological and premature aging regarding IGF‐1 plasma levels. Thus, while this hormone was significantly downregulated in the plasma of progeroid mice (again, corroborating the results obtained with the SomaScan Assay), an increase, albeit not statistically significant, in IGF‐1 levels was detected in the plasma of old mice compared to young mice.

### 2.3 Functional enrichment and disease analysis of progeroid plasma proteome

**FIGURE 3:**
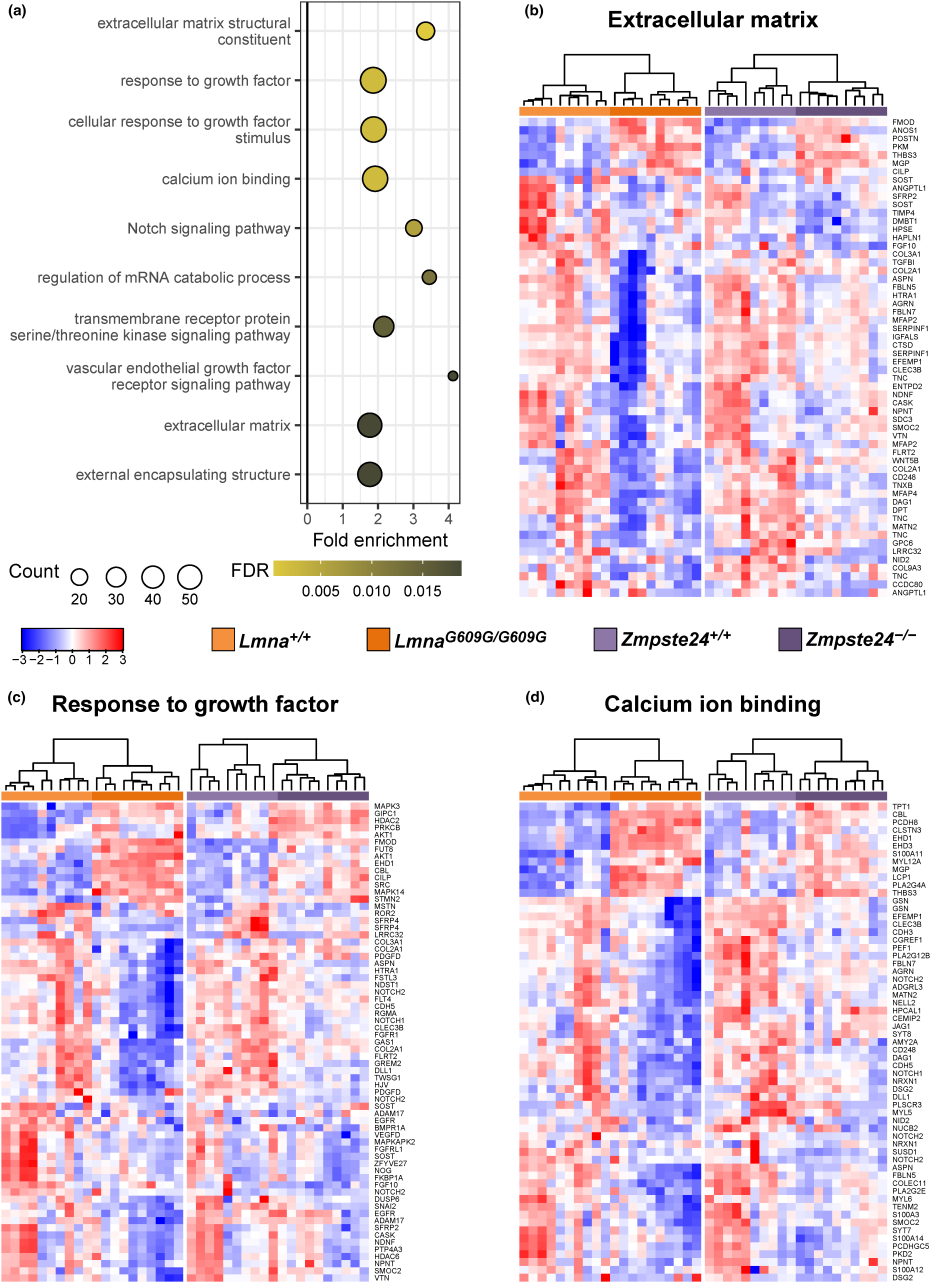
The pathways and gene sets most significantly enriched in the plasma proteome of progeroid mice exhibit a similar pattern across *Lmna* ^*G609G/G609G*^ and *Zmpste24* ^*−/−*^ mouse models. (a) Top 10 most significant enriched pathways in differentially expressed proteins between progeroid and wild‐type mice. The fold enrichment and number of proteins for each pathway are provided. (b–d) Heatmaps and hierarchical clustering of *Lmna* ^*G609G/G609G*^ and *Zmpste24* ^*−/−*^ mice for genes involved in extracellular matrix (GO:0031012; b), response to growth factor (GO:0070848; c) and calcium ion binding (GO:0005509; d). Clustering was carried out for each model (*Lmna* and *Zmpste24*) independently. Color key depicts the scaled expression for the three heatmaps for both models jointly considered. Expression scaling was independently performed for each gene set.

To determine the biological significance of the changes found in the plasma proteome of progeroid mouse models, we queried the Gene Ontology (GO) database to search for patterns of functional enrichment. To this end, we first performed an over‐enrichment analysis with the top 500 aptamers resulting from the overall comparison between progeroid and control mice. The pathways and gene sets enriched (FDR <0.05) included “extracellular matrix (ECM) structural constituent”, “response to growth factor”, “calcium ion binding”, “Notch signaling pathway” and “vascular endothelial growth factor receptor signaling pathway”, among others (Figure 3a). Notably, a closer inspection of the group of genes related to the top enriched extracellular matrix (Figure 3b), response to growth factor (Figure 3c) and calcium ion binding (Figure 3d) gene sets showed similar expression patterns in *Lmna* ^*G609G/G609G*^ and *Zmpste24* ^*−/−*^ mice, albeit with milder differences compared to controls in the latter. When performing the same type of analysis for each mouse model separately, similar pathways and gene sets were found to be significantly enriched in *Lmna* ^*G609G/G609G*^ mice (Figure S3a). Given the lower number of DE proteins found in *Zmpste24* ^*−/−*^ mice, only those gene sets mostly related to ECM were significantly enriched in this mouse model (Figure S3b). Nevertheless, although not meeting the statistical significance criteria, other gene sets enriched in *Lmna* ^*G609G/G609G*^ mice (such as “heart morphogenesis” and “Notch signaling”) also showed a differential pattern of expression in *Zmpste24* ^*−/−*^ versus wild‐type mice (Figure S3c–f). These enriched gene sets are consistent with the phenotype of progeroid mice, which mainly exhibit defects in mesenchyme‐derived tissues, including the cardiovascular system, where the ECM is a central element, in addition to growth retardation (Mariño et al., 2010; Osorio et al., 2011).

**FIGURE 4:**
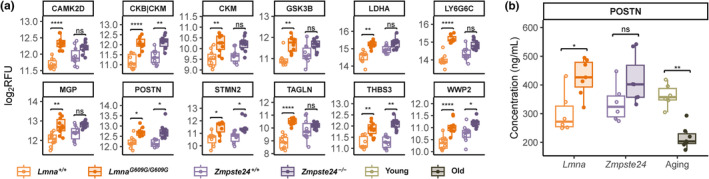
Cardiovascular disease‐related plasma proteins upregulated in *Lmna* ^*G609G/G609G*^ and *Zmpste24* ^*−/−*^ mice. (a) Boxplots of protein levels expressed as log_2_RFU for each biological replicate for significantly upregulated proteins related to cardiovascular disease in *Lmna* ^*G609G/G609G*^ and *Zmpste24* ^*−/−*^ mouse models compared to their corresponding wild‐type controls. (b) Plasma protein concentration of POSTN, measured by ELISA, in *Lmna* ^*+/+*^ (*n* = 6), *Lmna* ^*G609G/G609G*^ (*n* = 7), *Zmpste24* ^*+/+*^ (*n* = 6), *Zmpste24* ^*−/−*^ (*n* = 7), young (*n* = 6) and old (*n* = 7) mice. CAMK2D, calcium/calmodulin dependent protein kinase II delta; CKB, creatine kinase, brain isoform; CKM, creatine kinase, muscle isoform; GSK3B, glycogen synthase kinase 3 beta; LDHA, lactate dehydrogenase A; LY6G6C, lymphocyte antigen 6 family member G6C; MGP, matrix Gla protein; POSTN, periostin; STMN2, stathmin 2; TAGLN, transgelin; THBS3, thrombospondin 3; WWP2, WW domain‐containing protein 2 (also known as E3 ubiquitin‐protein ligase WWP2 or atrophin‐1‐interacting protein 2).

To explore potential associations between the plasma protein profile of our mouse models and the clinical manifestations of HGPS, we first queried the Disease Ontology database (Schriml et al., 2012). This analysis did not show any significant enrichment of genes belonging to a particular disease (data not shown), highlighting the profound and global dysregulation of the progeroid plasma proteome profiled herein. Interestingly, a bibliographic inspection of the top 10 most significantly DE plasma proteins in progeroid mice revealed that some of these proteins are involved in CVD, which is actually the main cause of death in children with HGPS (Gordon, Campbell, et al., 2018). This is the case of FMOD, whose expression is upregulated in clinical and experimental heart failure (Andenæs et al., 2018) or MFAP4, a protein whose abrogation in mice worsens cardiac function upon chronic pressure overload (Dorn et al., 2021) (Figures 1b and 2d). Based on these observations—and considering that one of the main goals of plasma proteome analysis is the identification of potential disease biomarkers or therapeutic targets—we decided to explore whether other proteins implicated in CVD were present among the set of upregulated proteins in at least one of the progeroid mouse models, given that most of the DE proteins in these mice were downregulated compared to their wild‐type controls. To this end, we performed a manual search based on the literature that allowed us to identify a list of 12 proteins which have been related to CVD and were significantly upregulated in the progeroid mouse models, especially in *Lmna* ^*G609G/G609G*^ mice, compared to wild‐type controls (Figure 4a and Table S3). Among these, we next validated by ELISA, and compared with those of naturally‐aged mice, the plasma levels of POSTN (periostin), a secreted matricellular cytokine upregulated in the heart following acute myocardial infarction (Kaur et al., 2016). As shown in Figure 4b, these experiments confirmed the increased presence of POSTN in the plasma of progeroid mice revealed by the SomaScan Assay. Conversely, this cytokine was significantly decreased in the plasma of old mice in comparison to young mice, again evidencing a difference in the secretome of progeroid and naturally‐aged mice.

### 2.4 “Old” plasma protein signature in premature aging models

In order to establish robust biomarkers of aging and age‐related disease that allow the monitorization of potential anti‐aging interventions, previous studies have developed epigenetic‐based clocks to estimate the biological age in premature aging syndromes (Horvath et al., 2018; Maierhofer et al., 2017). On this basis, we hypothesized that the plasma proteome may also recapitulate the age acceleration observed in progeria, given that proteomic clocks have already been used to accurately predict chronological age in physiological conditions (Lehallier et al., 2019).

**FIGURE 5:**
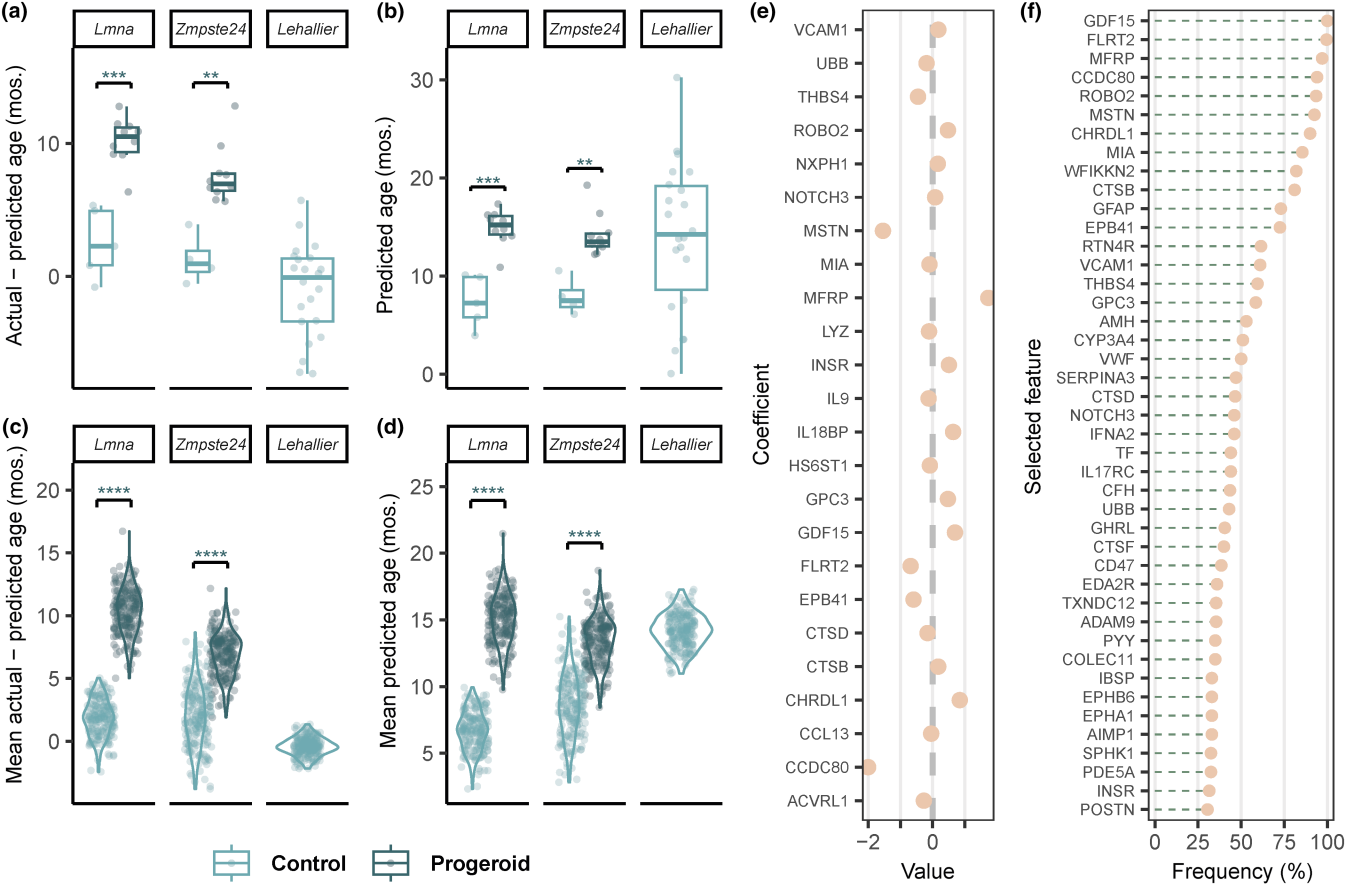
Proteomic clock from plasma samples predicts accelerated aging in progeroid mice. Two hundred random samplings were carried out in the control samples to obtain 200 different proteomic clocks, each of which was used to predict the age of the remaining healthy controls and of the progeroid mice. (a) Boxplot of the age gap determined for each group based on the prediction of one proteomic clock randomly selected from the 200 clocks developed. (b) Boxplot of the predicted age for each group by one clock randomly selected from the 200 clocks developed. (c) Violin plot of the age gap determined for each group based on the predictions of all 200 clocks developed. (d) Violin plot of the predicted age for each group by the 200 clocks developed. (e) Representative selection of proteins in a single clock and the magnitude of their positive or negative association with age acceleration. (f) Frequency of proteins present in at least 30% of the 200 clocks. mos., months.

To test this hypothesis, we used the existing SomaScan assay‐derived plasma proteomic datasets obtained from a cohort of 81 healthy mice ranging from 1 to 30 months of age (Lehallier et al., 2019). The availability of this data enabled us to develop a well‐calibrated aging clock that predicted the age of healthy controls with only a mean overestimation of 2 months (Figures 5a,b and S4). Remarkably, when we assessed the predicted age of progeroid mice, marked median age gaps—differences between chronological and predicted biological age—were found (10 and 7 months in the *Lmna* ^*G609G/G609G*^ and *Zmpste24* ^*−/−*^ groups, respectively) (Figure 5a). Thus, according to the plasma proteomic clock, the biological age in *Lmna* ^*G609G/G609G*^ and *Zmpste24* ^*−/−*^ progeroid mice was slightly over 15 and 13 months, respectively (Figure 5b). Given the low number of healthy wild‐type controls in our dataset to include in the least absolute shrinkage and selection operator (LASSO) regressions when generating the prediction model, we devised a sampling‐without‐replacement approach to verify our results by reducing sampling bias (Figure 5c,d). Further exploration of the plasma proteomic clock showed that well‐established age‐related proteins, such as growth differentiation factor 15 (GDF‐15), fibronectin leucine rich transmembrane protein 2 (FLRT2), growth differentiation factor‐8 (also known as “myostatin” [MSTN]) or coiled‐coil domain containing 80 (CCDC80) were frequently selected as predictors (Figure 5e,f).

To determine whether aging‐related proteins account for the main differences found between the plasma proteome of progeroid and control mice, we conducted a differential expression analysis on healthy mice with similar age to the ones predicted for the wild‐type and the progeroid groups. According to the data reported by Lehallier et al., and limiting the analysis to the 1156 aptamers determined in their study, only 67 were found to differ between healthy mice of the predicted ages, whereas this comparison raised up to 486 aptamers in the progeroid group versus controls. Nevertheless, this analysis revealed a relatively high overlap between healthy‐aged and progeroid mice, given that 47.8% (32 out of 67) of the aptamers reported in these comparisons were shared.

Therefore, the plasma proteomic clock revealed that the biological age in our progeroid models is significantly higher than their actual chronological age. However, even though there seemed to exist an overlap with physiological aging, the great increase in the number of DE proteins concomitant of the progeroid group suggests that, in addition to aging‐related proteins, there might be additional disease‐specific players in the development of the progeroid phenotype.

## 3 DISCUSSION

Circulating proteins have emerged as promising biomarkers of aging and disease given the reduced invasiveness involved in blood sample extraction and their potential to reflect whole‐body health status (Sebastiani et al., 2021; Tanaka et al., 2018; Williams et al., 2019). Moreover, plasma proteins have also been identified as causal agents in the development of diverse pathologies, as well as pro‐ and anti‐aging effectors (Katsimpardi et al., 2014; Villeda et al., 2011). In this study, we analyzed the plasma proteome of two progeroid mouse models, *Lmna* ^*G609G/G609G*^ and *Zmpste24* ^*−/−*^, using the SomaScan aptamer technology. To the best of our knowledge, this is the first large‐scale analysis performed in progeroid mice with the aim of better understanding HGPS disease and its relationship with aging. We had access to the currently latest version (v4.1) of the SomaScan platform, with 7291 aptamers (targeting 6388 unique proteins of interest) analyzed. Although this array was originally designed to analyze human proteins, previous versions of this technology have been successfully used before for the discovery of new biomarkers in mouse samples (Coenen‐Stass et al., 2015; Lehallier et al., 2019). Furthermore, validation analyses with mouse plasma samples were conducted by the company to determine the quality of each aptamer for detecting mouse proteins with the version v4.1 of the assay, which was employed in the study herein. Notably, 6379 aptamers (87.5%) in this assay were assigned high‐ or medium‐quality labels, highlighting the suitability of this technique for obtaining a global understanding of the plasma proteome from mouse models (SomaLogic, Inc. 2022). Of note, however, significant structural differences between human and mouse orthologs may mask the detection of certain proteins, which might have been considered as “not differentially” expressed in our study. Despite this limitation, a large number of proteins (2475) were found to be DE in the plasma of progeroid mice compared to controls, although the magnitude of change was modest in most cases. Interestingly, similar observations have been reported when analyzing the changes in plasma proteins with age (Lehallier et al., 2019; Sebastiani et al., 2021). Remarkably, the number of DE proteins was much higher in *Lmna* ^*G609G/G609G*^ than in *Zmpste24* ^*−/−*^ mice. These results may be consistent with some of the phenotypic differences between *Lmna* ^*G609G/G609G*^ and *Zmpste24* ^*−/−*^ mice. Although the survival for both mouse models has been improved over the years through the optimization in feeding and housing conditions, the impact of this refinement has been more pronounced in *Zmpste24* ^*−/−*^ mice (Bárcena et al., 2018). Thus, *Zmpste24* ^*−/−*^ mice live longer and show a lower mortality rate within the late life stage compared to *Lmna* ^*G609G/G609G*^ mice (Bárcena et al., 2018). Furthermore, a more severe cardiovascular phenotype has been reported in *Lmna* ^*G609G/G609G*^ mice compared to *Zmpste24* ^*−/−*^ mice (Benedicto et al., 2021). Nevertheless, there was a high correlation between the differences from controls observed in the plasma proteome of both progeroid mouse models, which share more than 90% of the significantly DE aptamers, suggesting similar underlying alterations, although different in magnitude. Likewise, one of the most striking findings of our study is the large number of proteins that were downregulated in the plasma of progeroid mice versus their controls with respect to those that showed upregulation. Our results are consistent with the greater proportion of proteins that showed lower levels in plasma samples from patients with HGPS compared to their controls in the only multiplex assay performed to date with human samples, where 90 proteins were analyzed (Gordon, Campbell, et al., 2018).

To gain insight into the potential biological processes that underlie the HGPS phenotype, we performed a functional enrichment analysis of the progeroid plasma proteomic signature. This analysis identified the “ECM structural constituent” as the most significantly enriched gene set in progeroid plasma samples. Indeed, among the top 10 list of DE proteins we found several proteins (PGM5, DPT, FMOD, and MFAP4) that play a role in ECM assembly, repair and remodeling‐related processes (Andenæs et al., 2018; Kanaan et al., 2022; Molt et al., 2014). This result is in agreement with previous studies showing a profound downregulation of ECM components in mouse fibroblasts derived from the progeroid Lmna∆9 mice (Hernandez et al., 2010). Indeed, we have previously reported that the proliferation defects of adult fibroblasts derived from *Zmpste24* ^*−/−*^ mice were rescued by the presence of wild‐type ECM (de la Rosa et al., 2013). Moreover, in this previous study, we demonstrated in vivo the relevance of cell‐extrinsic factors in the correction of the progeroid phenotype, which was completely reverted by the generation of a mosaic mice where progeroid Zmpste24‐deficient cells coexisted in similar proportions with Zmpste24‐proficient cells (de la Rosa et al., 2013). Notably, the gene expression of one of the proteins that was most downregulated in the secretome of our progeroid mice, DPT, was previously shown to be reduced in fibroblasts from patients with HGPS, where a profound deregulation in ECM composition was also observed (Csoka et al., 2004; Wang et al., 2006). Not surprisingly, the nuclear abnormalities characteristic of HGPS, caused by the perturbation of the main component of the nuclear scaffold, lamin A, have been related to alterations in nuclear mechanical properties that directly impact on the cytoskeleton and cellular functions (Vidak & Foisner, 2016). Thus, the combination of the reported defects in mechanotransduction, together with the observed alterations in the protein expression profile of ECM components, is consistent with the most prevalent pathologies of HGPS affecting tissues of a mesenchymal origin (Hennekam, 2006). Likewise, ECM damage has been largely associated with an aging phenotype but, most relevant, it has also been described that ECM alterations may contribute to the aging process (López‐Otín et al., 2023).

Our plasma proteome profiling also revealed an enrichment in proteins related to the cellular response to growth factors. Notably, one of the classic features of the progeroid phenotype is growth retardation, which is observed both in children suffering from HGPS and in progeroid mouse models (Gordon et al., 2014;Mariño et al., 2010; Osorio et al., 2011). Among these factors, IGF‐1 was in the top 10 list of the most significantly downregulated proteins, which is in agreement with previous studies performed with *Lmna* ^*G609G/G609G*^ and *Zmpste24* ^*−/−*^ mice (Mariño et al., 2010; Osorio et al., 2011). Indeed, administration of IGF‐1 to *Zmpste24* ^*−/−*^ mice leads to a delayed phenotype and longer life expectancy (Mariño et al., 2010). Although numerous studies with model organisms have evidenced that the attenuation of the somatotrophic growth hormone [GH]/IGF‐1 pathway increases lifespan in physiological aging, extremely low IGF‐1 levels may become deleterious, as it happens in progeroid mice (Lopez‐Otin et al., 2013). Despite the fact that IGF‐1 is not usually downregulated in patients with HGPS (Merideth et al., 2008), which highlights the inherent differences between humans and preclinical models, the exploration of this gene set and related pathways may lead to new clues to unveil the molecular mechanisms underlying the reduced growth and poor weight gain associated with HGPS. Functional enrichment analysis also revealed other pathways whose alterations were reflected in the plasma of progeroid mice, such as proteins involved in calcium ion binding. Calcium is an essential second signaling messenger that plays a key role in numerous physiological processes, including muscle contraction and growth, as well as enzyme activity regulation (Tu et al., 2016). In our study, we found a strong upregulation of calcium/calmodulin dependent protein kinase II delta (CAMK2D), a protein that has been related to the onset of heart failure in addition to its well‐known functions in calcium signaling transduction, cytoskeleton organization, and protein secretion (Backs et al., 2009; Zhang et al., 2003). We also found alterations in other calcium‐dependent proteins, such as COLEC11, a lectin that plays an important role in innate immunity through the activation of the complement pathway (Hwang et al., 2017). Of note, previous studies have related progerin expression with altered signaling in calcium‐related pathways (Fafián‐Labora et al., 2021; Wang et al., 2020). Interestingly, endoplasmic reticulum stress has been identified as a driver of the vascular pathology in HGPS, being this stress response affected by calcium homeostasis (Groenendyk et al., 2021; Hamczyk et al., 2019).

In addition to providing insights into biological processes that may account for the progeroid phenotype, the goal of the plasma proteome profiling herein is to identify potential HGPS biomarkers or targetable proteins relevant for the disease that could be monitored by means of a minimally invasive procedure—which is especially relevant in the context of patients with progeria, owing to their very frail/debilitated status. In this regard, an immunoassay has recently been optimized to detect progerin in the plasma of patients with HGPS as a valuable method to monitor clinical outcomes derived from progerin‐targeted therapies (Gordon et al., 2023). On this basis, given that CVD is the main cause of premature death in these patients, we performed a manual search in the literature for CVD‐related biomarkers among the pool of plasma proteins that were significantly upregulated in progeroid mice, considering that they represented a much smaller proportion than those that were downregulated. This search allowed us to identify 12 proteins related to CVD that were significantly upregulated in *Lmna* ^*G609G/G609G*^, the mouse model that better mimics the vascular phenotype of patients with HGPS. Nevertheless, several of these proteins were also upregulated in *Zmpste24* ^*−/−*^ mice. Among these proteins, creatine kinase‐MB (CK‐MB), an isoenzyme commonly used for the clinical diagnosis of acute myocardial infarction (Ghormade et al., 2014) was also found to be upregulated in the plasma of children with HGPS compared to controls, although the levels fell within the normal range of the assay (Gordon, Campbell, et al., 2018). Likewise, other proteins, such as lactate dehydrogenase A (LDHA), another isoenzyme previously used in the diagnosis of myocardial infarction and also involved in the proliferation of vascular smooth muscle cells (Kim et al., 2017) have been associated with arterial stiffness, one of the main phenotypes in HGPS pathology (Zhu et al., 2022). Besides, proteins related to the above‐mentioned enriched pathways found in the plasma proteome of progeroid mice, such as FMOD and CAMK2D, have also been related to heart failure (Andenæs et al., 2018; Zhang et al., 2003).

As a further attempt to explore the phenotype information contained in the progeroid plasma proteomic signature, we sought to evaluate its usefulness as aging clock, given that accelerated aging is the hallmark of HGPS. Classically, chronological age predictors rely on the information provided by several molecular biomarkers or clinical data of individuals to inform about their true biological age or the expected time to an event, generally death. The advent of recent high‐throughput techniques has enabled the use of data from thousands of markers across multiple biological layers. This has led to the development of multiple aging clocks built on different omics data, including epigenomic, transcriptomic, proteomic, metabolomic, glycomic, and microbiome‐based aging clocks (Rutledge et al., 2022). Biological age predictors based on the plasma proteome have the advantage of extracting information from a fluid whose composition depends on the activity of multiple organs and tissues throughout the body, thereby providing a measure of the overall health status of the organism. Additionally, the closer connection of proteins with biological mechanisms makes proteomic clocks more interpretable and many proposed predictor biomarkers constitute treatable targets (Johnson et al., 2021), providing a direct link to the aging process.

Previous attempts have been made to predict biological age from samples of patients with HGPS. Thus, an epigenomic clock predicted a higher age in fibroblasts from skin HGPS biopsies and two additional studies assigned an older age to cell lines derived from affected patients based on the same transcriptomic datasets (Fleischer et al., 2018; Horvath et al., 2018; Meyer & Schumacher, 2021). In our study, we harnessed cross‐sectional plasma proteomic data from a mouse cohort ranging from 1 to 30 months of age to develop an accurate chronological age predictor based on machine learning. We tested this aging clock on wild‐type controls of both *Lmna* ^*G609G/G609G*^ and *Zmpste24* ^*−/−*^ mouse models. The model yielded a small overestimation of 2 months above their mean true age, although this effect can be explained in terms of regression‐to‐the‐mean phenomena (Barnett, 2004). By contrast, this model predicted an age acceleration of 10 and 7 months when applied to *Lmna* ^*G609G/G609G*^ and *Zmpste24* ^*−/−*^ mice, respectively. This result supports the finding that progeroid mice exhibit an aged plasma proteomic signature. In addition, the greater biological age predicted on *Lmna* ^*G609G/G609G*^ mice is consistent with the shorter lifespan compared to *Zmpste24* ^*−/−*^ mice (Bárcena et al., 2018). Nevertheless, the age acceleration observed in these mouse models could be strongly dependent on the sampling age prior to their potential deaths. Thus, it has been shown that age acceleration does not necessarily follow a constant rate given that the expression of certain genes changes linearly while others follow nonlinear patterns with age (Schaum et al., 2020). The same can be applied to the plasma proteome, which changes over time in a nonlinear fashion (Lehallier et al., 2019). Thus, it is reasonable to speculate that systemic age acceleration also exhibits an undulating pattern, with the characteristic accelerated aging process interspersed with periods of slower aging. Regarding these two models of premature aging, it is possible that a bigger age gap could be predicted if plasma samples would have been gathered at a time point closer to their median age of death. However, it is more likely to conceive that other disease‐specific related factors, not captured by aging clocks, could play a role in the organismal deterioration of HGPS mouse models, given the great number of DE proteins detected in progeroid mice that are not shared with healthy mice of the same predicted ages.

**FIGURE 6:**
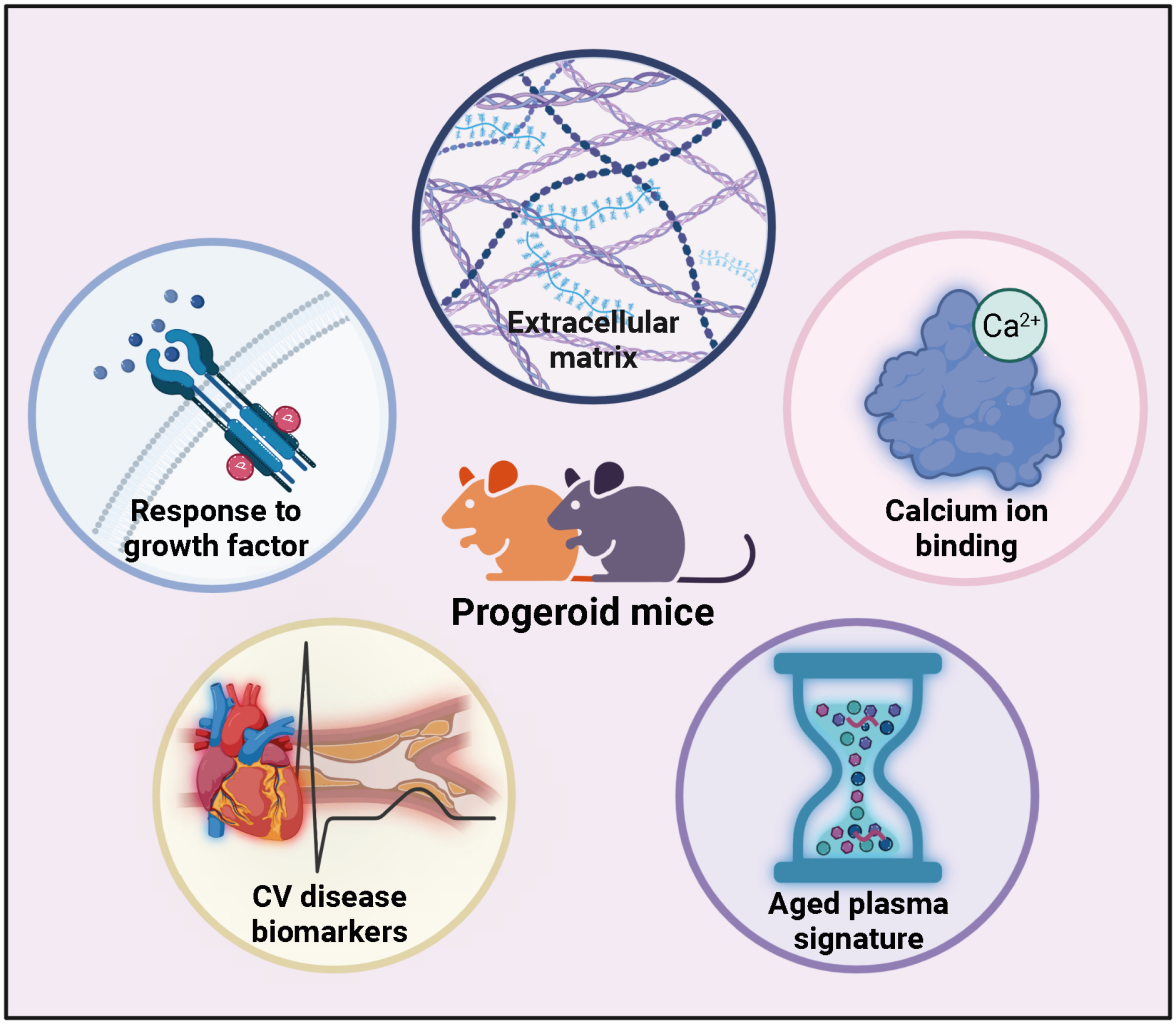
Overview of the main results derived from this study. Functional enrichment analysis of the top dysregulated proteins in *Lmna* ^*G609G/G609G*^ and *Zmpste24* ^*−/−*^ progeroid mouse models yielded sets of proteins involved in the extracellular matrix (ECM), response to growth factors and calcium ion binding. A manual review of the literature identified several upregulated proteins associated with cardiovascular disease (CVD). Additionally, publicly available plasma proteome data were leveraged to build an accurate chronological age predictor, which reported age acceleration in the plasma of *Lmna* ^*G609G/G609G*^ and *Zmpste24* ^*−/−*^ mice.

In conclusion, we have performed a global analysis of the plasma proteome of two mouse models of HGPS, which has allowed us to provide the first progeroid plasma proteomic signature, opening a broad field for future investigation. This analysis has shed light on some of the key biological processes that may underlie the progeroid phenotype and provided a better understanding of the similarities (and differences) between two of the mouse models more frequently used in the study of premature aging, as well as their commonalities with the physiological aging process. More importantly, we have identified potential plasma HGPS biomarkers that may help to track the progress of the disease or become *druggable* targets (Figure 6). Further research is needed to uncover whether some of the plasma proteins herein discovered may play a causal role in the development of the pathological phenotype, and, more important, their validation in affected patients, given the obvious limitations of preclinical models.

## 4 EXPERIMENTAL PROCEDURES

### 4.1 Animal samples

All animal procedures were performed in accordance with institutional guidelines and were reviewed and approved by the Research Ethics Committee of the University of Oviedo (Oviedo, Spain) and the *Consejería de Desarrollo Rural y Recursos Naturales del Principado de Asturias* (PROAE 19/2019, PROAE 53/2019, PROAE 29/2020). *Lmna* ^*G609G/G609G*^ and *Zmpste24* ^*−/−*^ mouse models were previously generated in our laboratory (Osorio et al., 2011; Varela et al., 2005). *Lmna* ^*G609G/G609G*^ mice were backcrossed at least 10 generations to C57BL/6NCrl background. *Zmpste24* ^*−/−*^ mice were on a mixed C57BL/6NCrl‐C57BL/6J background. Naturally‐aged mice and young controls were obtained from Janvier Labs in a C57BL/6JRj background and housed in the animal facilities of the University of Oviedo for at least 2 months prior to sample extraction. All mice were maintained under a 12‐h light‐darkness cycle. Progeroid mice were fed ad libitum and daily provided with powdered moistened standard diet in the cage due to the mandibular problems and the inability to access food that *Lmna* ^*G609G/G609G*^ and *Zmpste24* ^*−/−*^ mice tend to develop over time.

For the proteomic studies, blood was obtained from 40 mice distributed in the following groups: *Lmna* ^*+/+*^, *Lmna* ^*G609G/G609G*^, *Zmpste24* ^*+/+*^, and *Zmpste24* ^*−/−*^ mice (*n* = 10 mice per group). Data for candidate proteins were validated by ELISA and additionally assessed in naturally‐aged mice. Samples were taken between 4.5 and 5 months of age for *Lmna* mice, around 6.5 months of age for *Zmpste24* mice and young controls, and 25 months of age for naturally‐aged mice. Blood collection was performed in the morning by cardiac puncture with mice under a surgical anesthetic plane with isoflurane. Mice were not fasted prior to blood extraction. Blood was collected in 1.5 mL tubes containing 5 μL of 0.5 M EDTA (pH 7.5). In order to obtain plasma, samples were centrifuged at 2200 **g** for 15 min at room temperature and immediately frozen on dry ice. Plasma samples were then stored at −80°C until use.

### 4.2 Proteomic studies

The proteomic profiles of plasma samples were studied using the SomaScan Assay v4.1 (SomaLogic, Inc.). In its latest version, this assay includes 7596 aptamers (SOMAmers) that detect 7288 human proteins. Neither Lamin A nor ZMPSTE24 are included in the set of proteins recognized by the assay. Data for 7596 aptamers were obtained and only those probes targeting human or mouse proteins with an assigned *Entrez* gene symbol were kept for further analysis. For one protein (ADAM metallopeptidase with thrombospondin type 1 motif 1 [ADAMTS1]) there were two available aptamers targeting the human and the mouse ortholog. Only the aptamer specific for the mouse protein was kept. No sample data were excluded.

Data normalization was performed by SomaLogic as previously described (Candia et al., 2022). Normalized data were used for differential expression analysis with *limma* (Ritchie et al., 2015). First, *limma* *voom* function was used for mean–variance modelling and then *lmFit* for linear modeling to assess differential expression per aptamer. Principal component analyses (PCA) revealed a batch effect, so it was blocked at the *voom* step. Additionally, both sex and cohort were included in the linear model to estimate global differences, as well as model‐ and sex‐specific differences. Aptamers with FDR <0.05 were considered as DE.

The top 500 significant aptamers were provided to *clusterProfiler* (Wu et al., 2021) to determine pathway over‐representation with a hypergeometric test, using as background the list of proteins targeted by the SomaScan assay. GO lists were used, and only those pathways with FDR <0.1 were selected.

### 4.3 Proteomic clock

An aging clock was developed by training a model with a cross‐sectional mouse cohort obtained from Lehallier et al., 2019. Additionally, given that the inherent batch effect could hinder age estimation, randomly sampled wild‐type controls for both *Lmna* ^*G609G/G609G*^ and *Zmpste24* ^*−/−*^ mice were included in the model to enable the inference of its effect.

Both datasets were intersected using only proteins that were common to both datasets at the gene name level. Additionally, aptamers that were mapped to the same gene name were dropped to avoid ambiguity. Given the low number of wild‐type mice in the progeroid cohorts, we performed 200 random samplings and derived a LASSO linear regression model with *glmnet* (Friedman et al., 2010) for each iteration (200 total iterations), which allowed us to robustly determine that the sampling process was not biasing the results. We established the sampling step to require at least three wild‐type controls from our models both in the training and in the inference sets, in order to control for the batch effect across the two datasets.

The differential analysis of physiological aging was carried out with 16 mice ranging from 3 to 6 months‐old, as young controls, and 20 ranging from 18 to 21 months‐old. The statistical analysis in *limma* was carried out as stated before. The FDR threshold was set at 0.1.

### 4.4 Enzyme‐linked immunosorbent assays (ELISA)

Commercial enzyme‐linked immunosorbent assay kits were used to evaluate the plasma levels of the following proteins: FMOD (LS‐F23356, LSBio), IGF‐1 (MG100, R&D Systems) and POSTN (MOSF20, R&D Systems). Assays were performed following the manufacturer's instructions.

### 4.5 Statistical analysis

ELISA data and proteomic clock predictions were analyzed with the Wilcoxon rank‐sum test (Mann–Whitney *U* test). Data in boxplots show the median log_2_RFU for each protein, with lower and upper hinges corresponding to the first and third quartiles, respectively, and whiskers extending from the hinge to the furthest value at most 1.5 times the interquartile range from the hinge. Statistical significance levels are indicated as follows: *FDR <0.05, **FDR <0.01, ***FDR <0.001, ****FDR <0.0001.

## AUTHOR CONTRIBUTIONS

Diego Quintana‐Torres, Alejandra Valle‐Cao, Sandra Freitas‐Rodríguez, and Francisco Rodríguez performed experimental work. Diego Quintana‐Torres, Alejandra Valle‐Cao, and Pablo Bousquets‐Muñoz analyzed data. Alicia R. Folgueras, Alejandro López‐Soto, and Carlos López‐Otín conceptualized the study, supervised research and data interpretation. Diego Quintana‐Torres, Alejandra Valle‐Cao, Pablo Bousquets‐Muñoz, Alejandro López‐Soto and Alicia R. Folgueras wrote the manuscript. Alejandro Lucia contributed to data interpretation and writing of the manuscript. Manuscript was approved by all authors.

## FUNDING INFORMATION

This work was supported by Ministerio de Ciencia e Innovación (RTI2018‐096479‐A‐I00, PDI2020‐118394RB‐100 and PID2021‐126372OB‐I00), and European Research Council (742067, DeAge, ERC‐2016‐ADG). D.Q.‐T. is recipient of an FPU fellowship from Ministerio de Ciencia e Innovación (Spain). A.V.‐C. and P.B.‐M. are supported by FPI fellowships from Ministerio de Ciencia e Innovación (SAF2017‐87811‐R, RTI2018‐096479‐A‐I00). The IUOPA is funded by the Asturian Government and Fundación Cajastur‐Liberbank.

## CONFLICT OF INTEREST STATEMENT

The authors declare no conflicts of interest.

## Supporting information


Figures S1‐S4 and Tables S1‐S3:

## Data Availability

The data that support the results of this study are available at https://zenodo.org/ (DOI: https://doi.org/10.5281/zenodo.8009521).
