# Supplementary material for: The secretome atlas of two mouse models of progeria
Source: Aging Cell. 2023 Aug 10;22(10):e13952. doi: 10.1111/acel.13952 (PMC10577534; doi:10.1111/acel.13952)
Supplement: Supplementary file 1 — Figures S1‐S4 and Tables S1‐S3: [file ACEL-22-e13952-s001.pdf]

Supporting Information

The secretome atlas of two mouse models of progeria

Diego Quintana-Torres[1,4#], Alejandra Valle-Cao[1,4#], Pablo Bousquets-Muñoz[1#], Sandra Freitas-Rodríguez[1], Francisco Rodríguez[1], Alejandro Lucia[2,3], Carlos López-Otín[1‡], Alejandro López-Soto[1,4‡] and Alicia R. Folgueras[1,4‡]

[1]Departamento de Bioquímica y Biología Molecular, Facultad de Medicina, Instituto Universitario de Oncología del Principado de Asturias (IUOPA), Universidad de Oviedo, Oviedo, Spain
[2]CIBER of Frailty and Healthy Aging (CIBERFES) and Instituto de Investigación 12 de Octubre (i+12), Madrid, Spain
[3]Faculty of Sport Sciences, Universidad Europea, Madrid, Spain
[4]Instituto de Investigación Sanitaria del Principado de Asturias (ISPA), Oviedo, Spain

[#]These authors have contributed equally
[‡]These authors share senior authorship

LIST OF CONTENTS

Figure S1 1

Figure S2 2

Figure S3 3

Figure S4 4

Table S1 5

Table S2 5

Table S3 6

REFERENCES 6

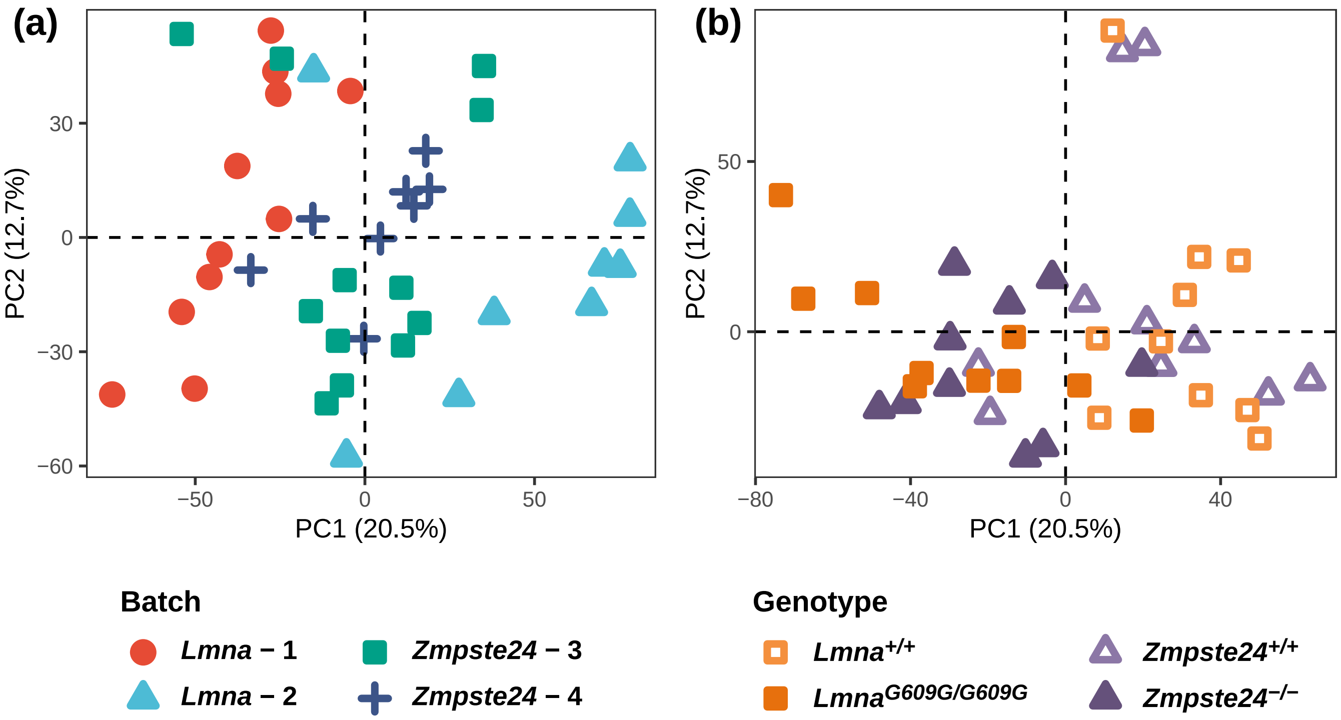

Figure S1. Principal Component Analysis (PCA) of the samples collected in this study. Samples are colored by batch (cohort) with raw data (a) or colored by genotype and model after batch effect correction (b).

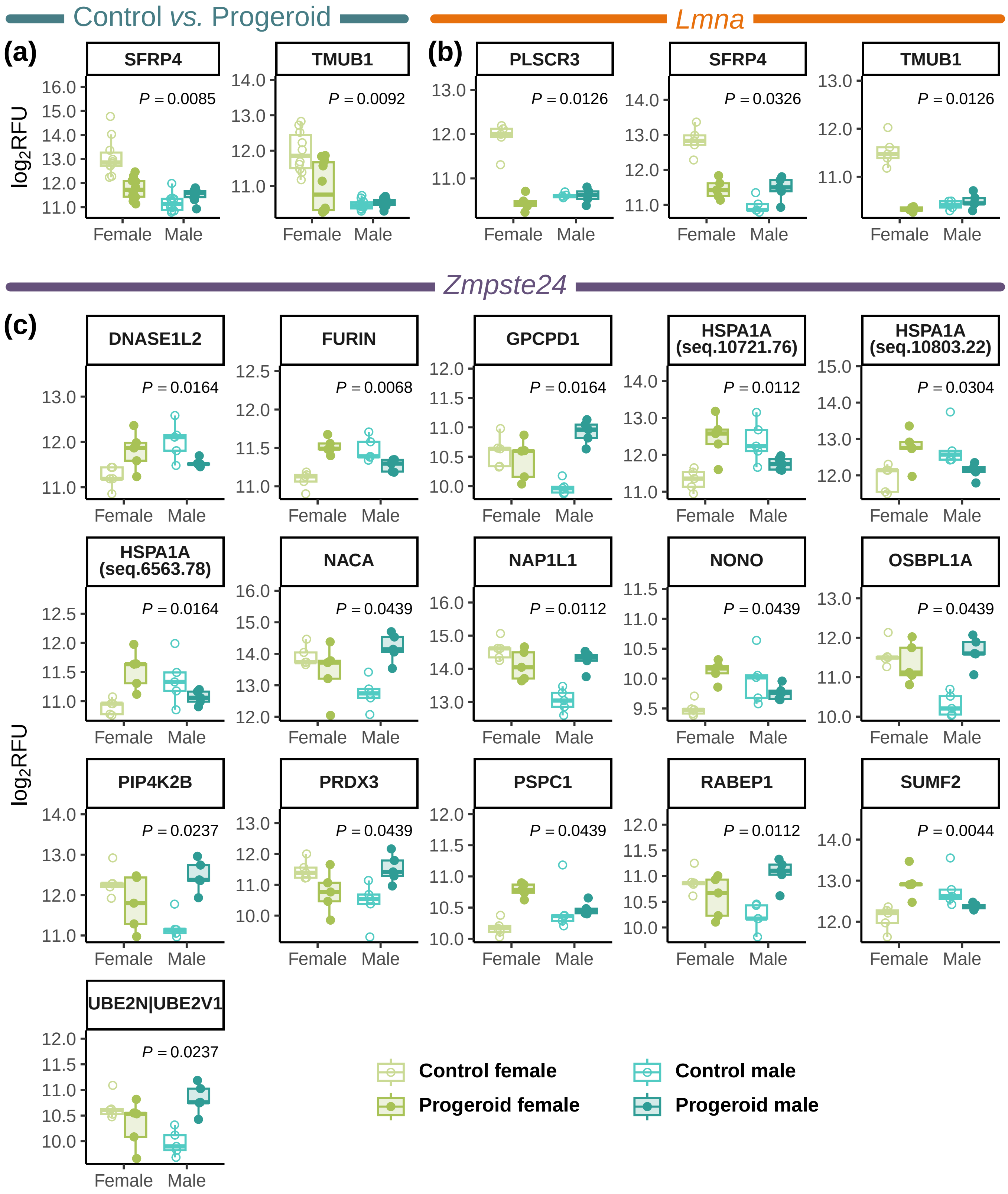

Figure S2. Analysis of sex-specific plasma proteome differences in progeroid mice. Boxplots of protein levels expressed as $\log_2$RFU for each biological replicate for the aptamers displaying a significant different behavior across sexes between control (n = 20, 10 female and 10 male) and progeroid mice (n = 20, 10 female and 10 male) (a), *Lmna*$^{+/+}$ (n = 10, 5 female and 5 male) and *Lmna*$^{G609G/G609G}$ mice (n = 10, 5 female and 5 male) (b), and *Zmpste24*$^{+/+}$ (n = 10, 5 female and 5 male) and *Zmpste24*$^{-/-}$ mice (n = 10, 5 female and 5 male) (c). Protein abbreviations: DNASE1L2, deoxyribonuclease 1 like 2; GPCPD1, glycerophosphocholine phosphodiesterase 1; HSPA1A, heat shock protein family A (Hsp70) member 1A; NACA, nascent-polypeptide-associated complex alpha polypeptide; NAP1L1, nucleosome assembly protein 1 like 1; NONO, non-POU domain-containing octamer-binding protein; OSBPL1A, oxysterol binding protein like 1A; PIP4K2B, phosphatidylinositol-5-phosphate 4-kinase type 2 beta; PLSCR3, phospholipid scramblase 3; PRDX3, peroxiredoxin 3; PSPC1, paraspeckle component 1; RABEP1, rabaptin, RAB GTPase binding effector protein; SFRP4, secreted frizzled related protein 4; SUMF2, sulfatase modifying factor 2; TMUB1, transmembrane and ubiquitin like domain containing 1; UBE2N, ubiquitin-conjugating enzyme E2 N; UBE2V1, ubiquitin-conjugating enzyme E2 V1.

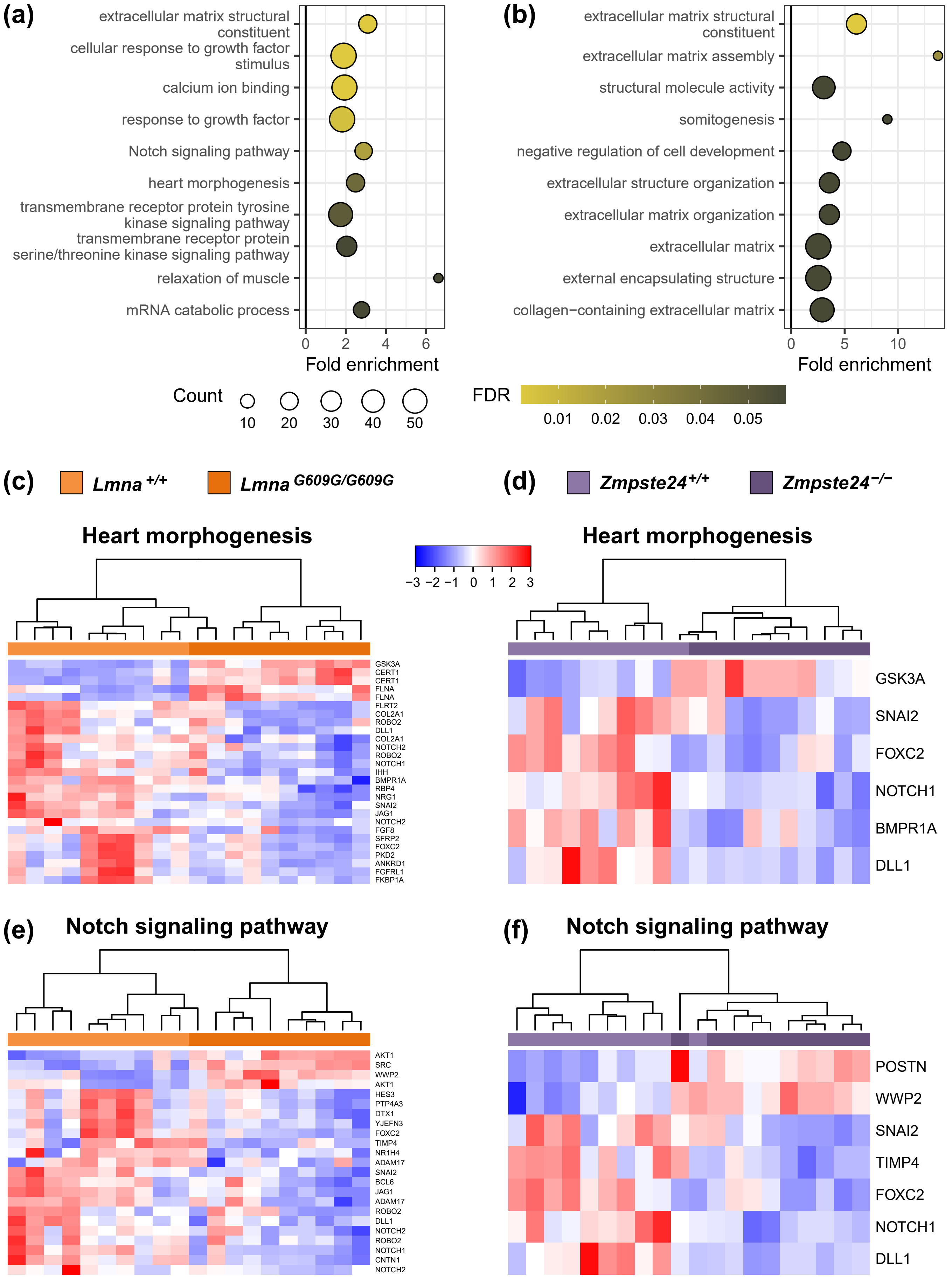

Figure S3. Pathway over-representation analysis in *Lmna*$^{G609G/G609G}$ and *Zmpste24*$^{-/-}$ mice. Top 10 most significant enriched pathways in DE proteins between *Lmna*$^{+/+}$ and *Lmna*$^{G609G/G609G}$ mice (a), and between *Zmpste24*$^{+/+}$ and *Zmpste24*$^{-/-}$ mice (b). The fold enrichment and number of proteins for each pathway are provided. (c-f) Heatmaps and hierarchical clustering of *Lmna*$^{G609G/G609G}$ (c, e) and *Zmpste24*$^{-/-}$ mice (d, f) for genes involved in heart morphogenesis (GO:0003007; c, d) and Notch signaling pathway (GO:0007219; e, f). For each heatmap, only the genes enriched in either *Lmna*$^{G609G/G609G}$ or *Zmpste24*$^{-/-}$ mice are accordingly depicted.

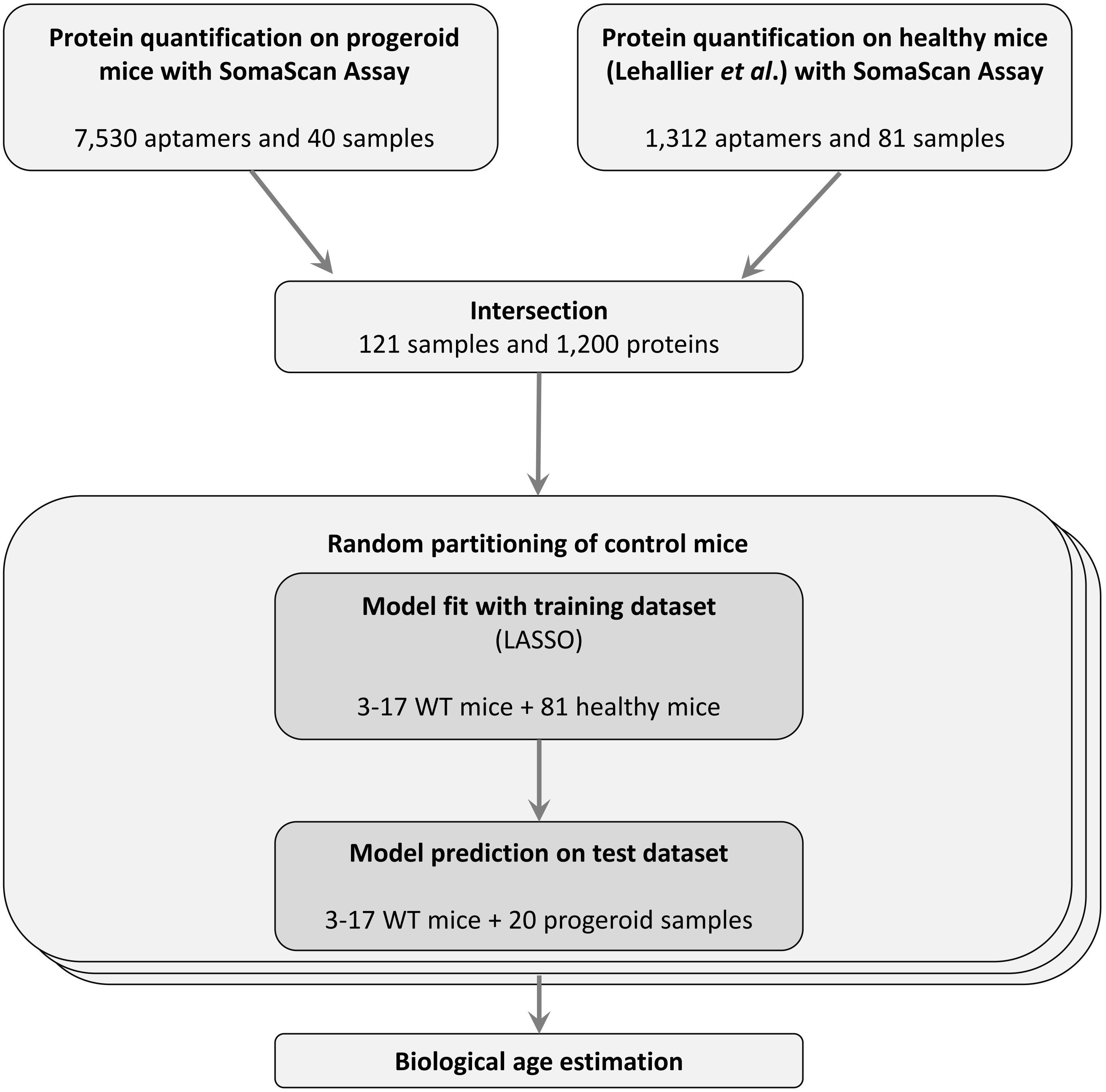

Figure S4. Workflow for the assessment of the chronological age in progeroid mice. Briefly, our dataset was merged with the one provided by Lehallier et al. and only the data for proteins unambiguously targeted by the same aptamer across the two datasets were kept. Only 1,200 unique proteins were left. To develop a chronological age predictor, we randomly partitioned the joint dataset for 200 iterations into a training and a test set. We constrained these partitions to keep at least 3 wild-type (WT) controls from our models in each set to correctly model the batch effect across the two original datasets and to assign all progeroid mice to the test set. The latter group was used to predict the chronological age of the progeroid samples as well as of the remaining WT controls. To ensure the consistency in our conclusions, we used the results from the 200 iterations to infer the biological age of progeroid mice.

Table S1. Top 10 most significant differentially expressed proteins in *Lmna*$^{G609G/G609G}$ mice versus wild-type controls.

Gene symbol	UniProt ID	Aptamer	log_2FC	FDR
PGM5	Q15124	seq.25117.17	2.19	1.21E-12
MFAP4	P55083	seq.5636.10	-2.41	1.21E-12
DPT	Q07507	seq.4979.34	-1.40	1.37E-10
ABR	Q12979	seq.21958.4	1.51	1.37E-10
IGF1	P05019	seq.2952.75	-1.25	1.37E-10
GLDN	Q6ZMI3	seq.20591.48	-1.69	1.19E-09
PCDH8	O95206	seq.6280.11	1.57	1.19E-09
H2AC1	Q96QV6	seq.22402.12	1.34	1.19E-09
RTN4R	Q9BZR6	seq.5105.2	-1.00	2.35E-09
COLEC11	Q9BWP8	seq.4430.44	-1.13	2.71E-09

Table S2. Top 10 most significant differentially expressed proteins in *Zmpste24*$^{-/-}$ mice versus wild-type controls.

Gene symbol	UniProt ID	Aptamer	log2FC	FDR
FMOD	Q06828	seq.6367.66	1.41	2.26E-08
DPT	Q07507	seq.4979.34	-0.88	3.01E-05
GLDN	Q6ZMI3	seq.20591.48	-0.99	2.94E-04
RTN4R	Q9BZR6	seq.5105.2	-0.64	2.94E-04
PGM5	Q15124	seq.25117.17	0.99	3.96E-04
PCDH8	O95206	seq.6280.11	0.90	5.73E-04
GREM2	Q9H772	seq.5598.3	-0.48	7.15E-04
COLEC11	Q9BWP8	seq.4430.44	-0.71	1.46E-03
PLA2G12B	Q9BX93	seq.9380.2	-0.71	1.79E-03
SCG3	Q8WXD2	seq.7957.2	-0.84	1.83E-03

Table S3. List of significantly upregulated proteins found in *Lmna*$^{G609G/G609G}$ and *Zmpste24*$^{-/-}$ mice plasma related to cardiovascular disease.

Gene symbol	UniProtID	Aptamer	References
CKM	P06732	seq.3714.49	(Ghormade et al. 2014; Moussa et al. 2013; Minamidate et al. 2022)
CAMK2D	Q13557	seq.3419.49	(Backs *et al*. 2009; Zhang *et al*. 2003; Ling *et al*. 2009)
**FMOD**	Q06828	seq.6367.66	(Andenæs et al. 2018)
GSK3B	P49841	seq.3236.12	(Woulfe *et al*. 2010)
LDHA	P00338	seq.15414.316	(Kim et al. 2017; Zhu et al. 2022; Ghormade et al. 2014)
LY6G6C	O95867	seq.6256.9	(Zhong *et al*. 2021)
MGP	P08493	seq.6520.87	(Zwakenberg et al. 2018; Jono et al. 2004; Buyukterzi et al. 2018; Malhotra et al. 2022)
POSTN	Q15063	seq.6645.53	(Shimazaki et al. 2008; Kaur et al. 2016)
STMN2	Q93045	seq.10900.272	(Ke *et al*. 2022)
TAGLN	Q01995	seq.9756.6	(Huang et al. 2018; Zhou et al. 2022)
THBS3	P49746	seq.8982.65	(Ukkat et al. 2023; Chen et al. 2022; Schips et al. 2019)
WWP2	O00308	seq.21670.52	(Chen *et al*. 2019; Ghormade *et al*. 2014)

REFERENCES

Buyukterzi Z, Can U, Alpaydin S, Guzelant A, Karaarslan S, Mustu M, Kocyigit D & Gurses KM (2018) Enhanced serum levels of matrix Gla protein and bone morphogenetic protein in acute coronary syndrome patients. J. Clin. Lab. Anal. 32, e22278. DOI: 10.1002/jcla.22278.

Chen H, Moreno-Moral A, Pesce F, Devapragash N, Mancini M, Heng EL, Rotival M, Srivastava PK, Harmston N, Shkura K, Rackham OJL, Yu W-P, Sun X-M, Tee NGZ, Tan ELS, Barton PJR, Felkin LE, Lara-Pezzi E, Angelini G, Beltrami C, Pravenec M,

Schafer S, Bottolo L, Hubner N, Emanueli C, Cook SA & Petretto E (2019) WWP2 regulates pathological cardiac fibrosis by modulating SMAD2 signaling. Nat. Commun. 10, 3616. DOI: 10.1038/s41467-019-11551-9.

Chen Y, Meng H, Meng X, Yan Z, Wang J & Meng F (2022) Correlation Between Low THBS3 Expression in Peripheral Blood and Acute Myocardial Infarction. Front. Biosci.-Landmark 27, 291. DOI: 10.31083/j.fbl2710291.

Huang L, Li L, Yang T, Li W, Song L, Meng X, Gu Q, Xiong C & He J (2018) Transgelin as a potential target in the reversibility of pulmonary arterial hypertension secondary to congenital heart disease. J. Cell. Mol. Med. 22, 6249–6261. DOI: 10.1111/jcmm.13912.

Jono S, Ikari Y, Vermeer C, Dissel P, Hasegawa K, Shioi A, Taniwaki H, Kizu A, Nishizawa Y & Saito S (2004) Matrix Gla protein is associated with coronary artery calcification as assessed by electron-beam computed tomography. Thromb. Haemost. 91, 790–794. DOI: 10.1160/TH03-08-0572.

Kaur H, Takefuji M, Ngai C y., Carvalho J, Bayer J, Wietelmann A, Poetsch A, Hoelper S, Conway SJ, Möllmann H, Looso M, Troidl C, Offermanns S & Wettschureck N (2016) Targeted Ablation of Periostin-Expressing Activated Fibroblasts Prevents Adverse Cardiac Remodeling in Mice. Circ. Res. 118, 1906–1917. DOI: 10.1161/CIRCRESAHA.116.308643.

Ke X, Guo W, Peng Y, Feng Z, Huang Y, Deng M, Wei M & Wang Z (2022) Investigation into the role of Stmn2 in vascular smooth muscle phenotype transformation during vascular injury via RNA sequencing and experimental validation. Environ. Sci. Pollut. Res. 29, 3498–3509. DOI: 10.1007/s11356-021-15846-7.

Ling H, Zhang T, Pereira L, Means CK, Cheng H, Gu Y, Dalton ND, Peterson KL, Chen J, Bers D & Brown JH (2009) Requirement for Ca2+/calmodulin–dependent kinase II in the transition from pressure overload–induced cardiac hypertrophy to heart failure in mice. J. Clin. Invest. 119, 1230–1240. DOI: 10.1172/JCI38022.

Malhotra R, Nicholson CJ, Wang D, Bhambhani V, Paniagua S, Slocum C, Sigurslid HH, Lino Cardenas CL, Li R, Boerboom SL, Chen Y-C, Hwang S-J, Yao C, Ichinose F, Bloch DB, Lindsay ME, Lewis GD, Aragam JR, Hoffmann U, Mitchell GF, Hamburg NM, Vasan RS, Benjamin EJ, Larson MG, Zapol WM, Cheng S, Roh JD, O'Donnell CJ, Nguyen C, Levy D & Ho JE (2022) Matrix Gla Protein Levels Are Associated With Arterial Stiffness and Incident Heart Failure With Preserved Ejection Fraction. Arterioscler. Thromb. Vasc. Biol. 42, e61–e73. DOI: 10.1161/ATVBAHA.121.316664.

Minamidate N, Takashima N & Suzuki T (2022) The impact of CK-MB elevation in patients with acute type A aortic dissection with coronary artery involvement. J. Cardiothorac. Surg. 17, 169. DOI: 10.1186/s13019-022-01924-5.

Moussa ID, Klein LW, Shah B, Mehran R, Mack MJ, Brilakis ES, Reilly JP, Zoghbi G, Holper E & Stone GW (2013) Consideration of a new definition of clinically relevant myocardial infarction after coronary revascularization: an expert consensus document from the Society for Cardiovascular Angiography and Interventions (SCAI). J. Am. Coll. Cardiol. 62, 1563–1570. DOI: 10.1016/j.jacc.2013.08.720.

Schips TG, Vanhoutte D, Vo A, Correll RN, Brody MJ, Khalil H, Karch J, Tjondrokoesoemo A, Sargent MA, Maillet M, Ross RS & Molkentin JD (2019) Thrombospondin-3 augments injury-induced cardiomyopathy by intracellular integrin inhibition and sarcolemmal instability. Nat. Commun. 10, 76. DOI: 10.1038/s41467-018-08026-8.

Shimazaki M, Nakamura K, Kii I, Kashima T, Amizuka N, Li M, Saito M, Fukuda K, Nishiyama T, Kitajima S, Saga Y, Fukayama M, Sata M & Kudo A (2008) Periostin is essential for cardiac healingafter acute myocardial infarction. J. Exp. Med. 205, 295–303. DOI: 10.1084/jem.20071297.

Ukkat J, Rebelo A & Trojanowicz B (2023) Angiogenetic transcriptional profiling reveals potential targets modulated in blood of patients with cardiovascular disorders. Vascular 31, 152–162. DOI: 10.1177/17085381211052379.

Woulfe KC, Gao E, Lal H, Harris D, Fan Q, Vagnozzi R, DeCaul M, Shang X, Patel S, Woodgett JR, Force T & Zhou J (2010) Glycogen Synthase Kinase-3β Regulates Post–Myocardial Infarction Remodeling and Stress-Induced Cardiomyocyte Proliferation In Vivo. Circ. Res. 106, 1635–1645. DOI: 10.1161/CIRCRESAHA.109.211482.

Zhong Y, Chen L, Li J, Yao Y, Liu Q, Niu K, Ma Y & Xu Y (2021) Integration of summary data from GWAS and eQTL studies identified novel risk genes for coronary artery disease. Medicine (Baltimore) 100, e24769. DOI: 10.1097/MD.0000000000024769.

Zhou J-J, Yang J, Li L, Quan R-L, Chen X-X, Qian Y-L, Huang L, Wang P-H, Li Y, Meng X-M, Chen X, Gu Q & He J-G (2022) Transgelin exacerbates pulmonary artery smooth muscle cell dysfunction in shunt-related pulmonary arterial hypertension. ESC Heart Fail. 9, 3407–3417. DOI: 10.1002/ehf2.14080.

Zwakenberg SR, van der Schouw YT, Vermeer C, Pasterkamp G, den Ruijter HM & Beulens JWJ (2018) Matrix Gla Protein, Plaque Stability, and Cardiovascular Events in Patients with Severe Atherosclerotic Disease. Cardiology 141, 32–36. DOI: 10.1159/000493006.
